# Hypokalaemic paralysis in an adult case of *Plasmodium vivax* malaria

**DOI:** 10.1186/1475-2875-12-111

**Published:** 2013-03-26

**Authors:** Sagar Sinha, Ananya Mukherji, Santwana Chandrakar

**Affiliations:** 1 Department of Medicine, Dr DY Patil Medical College, Hospital and Research Centre, Sector-5, Nerul, Navi Mumbai (New Bombay), Maharashtra, 400706, India

**Keywords:** Malaria, *Plasmodium vivax*, Hypokalaemia, Paraparesis

## Abstract

Hypokalaemia and its subsequent complications are more often seen in children rather than in adults and are more common with *falciparum* malaria. This is a case of a 26-year-old male with *Plasmodium vivax* malaria who had developed paraparesis secondary to hypokalaemia. His treatment involved correction of the potassium level as well as the treatment of malaria. Such an atypical manifestation of malaria in an adult has not been previously documented.

## Case presentation

A 26-year-old male presented at the hospital with complaints of high grade intermittent fever, with chills, for two days and the sudden onset of progressive weakness for a day. The fever was not accompanied by vomiting, diarrhoea or any other systemic complaints. The patient had been treated by a local doctor after his complete blood count screen had revealed *Plasmodium vivax* malaria (trophozoites in blood smear) with thrombocytopenia (platelets 56,000/mm^3^). He had been given cefixime 200mg po bid and artesunate 200mg po od for a day. The next day, he had suddenly developed gradually progressive lower limb weakness leading to an inability to stand up and walk. He denied any history of recent vaccinations, use of diuretics, any trauma or seizures. There was neither any history of a similar weakness in his past nor any family history of it. The patient denied any history of alcohol, tobacco or recreational drug use. A driver by occupation, he lived in an urban area. He denied any prior history of malaria.

On physical examination, his pulse was 74/min, regular; his blood pressure was 124/70 mm Hg and his abdominal examination revealed mild splenomegaly. His cardiac examination was unremarkable. The neurological examination showed bilateral lower limb flaccid paralysis (grade I power) with knee and ankle reflexes absent, with absent bilateral plantar reflexes. In the lower limbs, the muscle tone was reduced without any muscle tenderness. Lower limb sensations were intact. The power was 5/5 in upper limbs with no sensory deficit. The cranial nerves examination was normal. The patient was admitted to the hospital and his blood samples were sent for urgent tests for serum electrolytes. The results revealed a potassium value of 1.47 mEq/L. The ECG, correspondingly, showed flattened T waves and the presence of U waves.

**Figure 1:**
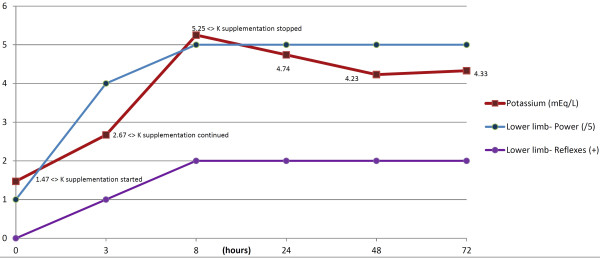
Correlation of the potassium value with the lower limb neurological examination findings over the course of hospitalization.

Intravenous potassium chloride was given for correcting the potassium level and, since it was a life-threatening hypokalaemia (<1.5 mEq/L), at a relatively rapid rate of 30 mEqs/hr. Repeat serum chemistry after three hours showed a potassium value of 2.67 mEq/L. There was some improvement in clinical signs of weakness and ECG. The rate of correction was subsequently slowed down to 20mEqs/hr. After a total infusion of 150 mEqs potassium IV, electrolyte tests repeated after 8 hours, showed a potassium value of 5.25 mEq/L. Potassium supplementation was then stopped. The correlation of the potassium value with the neurological examination and ECG is given in Table 1, also indicating the moments when potassium supplementation was carried out. The patient did not have any complaints after 8 hours of hospitalization. The repeat electrolytes tests done on the subsequent days revealed potassium in the normal range. The correlation of the potassium value with the lower limb neurological examination findings over the course of hospitalization is given in Figure 1.

**Table 1:** The correlation of the potassium value with the neurological examination and ECG

**Time**	**Day 1–0 hours**	**Day 1–3 hours**	**Day 1–8 hours**	**Day 2/3/4**
Potassium (mEq/L)	1.47	2.67	5.25	4.74/4.23//4.33
Potassium Supplementation	Started at 30 mEqs/hr for 3 hours	Continued at 20 mEqs/hr for 3 hours	Stopped	-
Total mEqs supplemented	0	90	150	-
ECG	Flattened T waves, U waves present	Flattened T waves present, U waves absent	Normal	Normal
Upper Limbs- Tone	Normal	Normal	Normal	Normal
Upper Limbs- Power	5/5	5/5	5/5	5/5
Upper Limbs- Reflexes	Normal (2+)	Normal (2+)	Normal (2+)	Normal (2+)
Upper Limbs- Sensory	Normal	Normal	Normal	Normal
Lower Limbs- Sensory	Normal	Normal	Normal	Normal
Lower Limbs- Tone	Hypotonia (flaccid)	Normal	Normal	Normal
Lower Limbs- Power	1/5	4/5	5/5	5/5
Lower Limbs- Reflexes (Knee and Ankle)	Absent (0)	Present (1+)	Present (2+), normal	Present (2+), normal
Plantar Reflex	Absent	Absent	Normal flexor response	Normal flexor response

Arterial blood gas, serum magnesium, serum calcium, blood sugar and renal function were within normal limits. The liver function tests showed mild indirect bilirubinemia. His urine test results were within the normal range. The patient also tested negative for dengue, leptospirosis and HIV. Total T4 was mildly elevated; however, TSH and Total T3 were normal. The summary of the blood investigations is given in Table 2.

**Table 2:** Summary of haematology results

	**Units**	**Reference range**	**Pre-admission**	**Day 1**			**Day 2**	**Day 3**	**Day 4**
Haemoglobin	gm/dL	11-18	11.5	11.1			10.8	11.4	
Total Leukocyte Count	K/uL	4-10	7.9	8.1			5.5	6.4	
Platelet Count	/uL	150,000-400,000	56,000	76,000			31,000	179,000	
Peripheral Smear			Trophozoites of *Plasmodium vivax*	Trophozoites of *Plasmodium vivax*			malarial parasite not seen	malarial parasite not seen	
Glucose-6-Phosphate Dehydrogenase								no deficiency seen	
				0 hrs	3 hrs	8 hrs			
Serum Electrolytes	mEq/L	Sodium: 135-148		141	141	143	140	138	139
		**Potassium: 3.7-5.3**		**1.47**	**2.67**	**5.25**	**4.74**	**4.23**	**4.33**
		Chloride: 100-108		110	110	109	107	107	107
Serum Calcium	mg/dL	8.5-10.1		9.2				9.4	
Serum Magnesium	mg/dL	1.7-2.3		2.3				2.1	
Serum Creatinine	mg/dL	0.6-1.3		0.8					0.8
Blood Urea Nitrogen	mg/dL	7-18		11					12
Bilirubin	mg/dL	Total: 0-1		1.5					
		Direct: 0–0.3		0.2					
		Indirect: 0–0.7		1.3					
Arterial Blood Gas				Normal					
HIV	ELISA						Non-Reactive		
Leptospirosis Antibody	IgM						Negative		
Dengue Rapid	NS-1 Antigen						Negative		
	IgM Antibody						Negative		
	IgG Antibody						Negative		
Thyroid Profile	mcg/dL	TSH: 0.4-5.3					4.94		
		Total T3: 69-205					129.97		
		Total T4: 4.5-12.6					13.7		

Note: Other investigations like liver enzymes, serum uric acid, serum proteins, serum albumin, blood sugars and urine (routine and microscopy) were within normal limits.

The patient received artesunate 120mg IV 12 hourly on day 1 followed by od dose for two days, ceftriaxone 1gm IV 12 hourly for three days and doxycycline 100mg po bid for five days. Injectibles were stopped on day 4 and he was put on artemether/lumefantrine 80/480mg po bid for three days; and to prevent a relapse, primaquine 7.5mg po bid was added for fourteen days after testing for glucose-6-phosphate dehydrogenase deficiency.

The patient was discharged home on day 5. He returned for a follow-up consultation two weeks later. He did not have any complaints, including fever, during that intervening period.

## Discussion

Around 1.5 million confirmed cases of malaria are reported from India annually. The current national policy recommends artemisinin combination therapy for *falciparum* cases and chloroquine plus primaquine for *vivax* cases. Oral artemisinin monotherapy is banned in India to prevent resistance. However, the policy also recommends prompt treatment of severe malaria with parenteral artemisinin derivatives or quinine [1]. In the region of greater Mumbai, malaria has a high prevalence of more than 1 case per 1,000 population [2]. Resistance to chloroquine has been steadily rising in this region as well [3]. As a result, across the region, for treatment of even *vivax* cases, artemisinin combination therapy has become quite popular, with an addition of doxycycline or clindamycin.

Dworak *et al.* stated that there was a progressive decrease in the sodium and potassium levels within 12 hrs of the parasite’s entry into the host [4]. Hypokalaemia in malaria has been observed in *falciparum* cases in children, where the underlying cause was proposed to be the correction of acidosis seen usually in severe cases of malaria [5]. A study from Gujarat, India, found hyponatremia and hypokalaemia to be more common in *falciparum* than *vivax* malaria [6]. Another study from Nigeria showed significantly lowered sodium and potassium levels in malaria infection [7]. In their analysis, Thanachartwet *et al.* found a high prevalence of hypokalaemia in Thai patients with malaria, and significant association of hypokalaemia with *vivax* infection, hyponatraemia and hypovolaemia. They postulated that the hypokalaemia was multifactorial, from a combination of intracellular translocation of potassium from extracellular fluid and urinary potassium loss [8].

Case reports have been published concerning motor weakness secondary to hypokalaemia in other infectious diseases in adults, namely chikungunya [9], dengue fever [10] and leptospirosis [11]. Periodic paralysis has also been reported in malaria wherein the combination of transient hyperkalaemia and rigors occurring during febrile episodes of malaria has been suggested as the underlying cause which precipitating muscular paralysis [12]. Although malaria has been found to be associated with various neurological and psychiatric complications, and neurological manifestations to anti-malarial drugs have been documented [13]; the authors could not find any literature documenting the association of such a type of motor weakness (hypokalaemic) with malaria.

There have been case reports of malaria causing Guillian Barre Syndrome [14,15], but since the patient’s recovery from the paraparesis was dramatic and complete within a few hours of potassium supplementation, it was easily excluded from the differential diagnosis and neither the EMG/NCV studies nor a lumbar puncture (for CSF analysis) was performed. Since the patient had only lower limb muscle weakness, without the involvement of any higher functions or cranial nerves and in the absence of upper motor neuron signs, MRI was not performed.

There was no obvious fluid loss since there was no report of vomiting or diarrhoea or any altered urine output. Increased catecholamine levels in response to stress of the infection and secondary insulin release may result in intracellular shift of potassium and hypokalaemia. Hyperventilation due to hyperpyrexia and respiratory alkalosis has also been suggested as a cause for hypokalaemia [16].

The patient’s magnesium levels were normal and T4 was mildly elevated. There was no strenuous exercise preceding the weakness thereby ruling out any glycogen storage disorder. Also, since there was no similar episode of weakness in the past or any family history of it, familial channelopathies were not included in the differential diagnosis and got no further workup.

Also, artemisinins can potentially cause hypokalaemia. They have been known to affect voltage-gated potassium currents, and their administration in patients of hypokalaemia has been known to prolong the QT-interval. Hara *et al.* examined the effect of artemisinins on ligand-gated potassium currents and found an inhibitory effect [17]. Development of immunity, increasing resistance to antimalarial drugs and indiscriminate use of anti-malarial drugs have also been proposed as the causes for malaria presenting with unusual features in endemic areas [18].

The authors believe that in this case the patient developed hypokalaemia most likely due to a distributional shift of potassium, which was potentiated by the drugs he received before his hospitalization. However, he did not have any similar response to the artemisinins after admission (artesunate IV) and after discharge (artemether po).

## Conclusion

Physicians, especially those in endemic areas of malaria, must consider motor weakness being caused by complications secondary to infectious diseases, especially a prevalent one like malaria. Potassium levels must be monitored in a case presenting with such symptoms.

Once the presence of hypokalaemia in a case has been established, the authors recommend the following approach:

– to determine its cause

– to fully evaluate and investigate the patient

– to treat the hypokalaemia (aggressively, if life-threatening) and its complications

– to treat the malaria

– to treat the underlying cause/trigger of the hypokalaemia.

A thorough neurological examination must be done to determine the extent of the weakness and its type (whether flaccid or spastic) and associated features. An ECG must be quickly obtained as well. The patient’s fluid status must be evaluated to rule out hypovolaemia and urine output must be monitored. Arterial blood gas should be tested to evaluate alkalosis, anion gap and bicarbonate levels. Magnesium and calcium deficiency must also be ruled out. Persistent weakness despite potassium correction and normalization of its values could warrant EMG/NCV, imaging and CSF tests. Thyroid profile should also be done to exclude periodic paralysis.

From the existing literature, it is clear that hypokalaemia and hyponatremia are now well documented phenomena in malaria patients. However, the actual incidence of hypokalaemia and its associated clinical manifestations may be highly underestimated. Further studies are required to determine its epidemiology and pathophysiology.

### Patient’s consent

Written informed consent was obtained from the patient for publication of this Case report and any accompanying images. A copy of the written consent is available for review by the Editor-in-Chief of this journal.

## Competing interests

The authors declare that they have no competing interests.

## Authors’ contributions

SS conceived of the study and drafted the manuscript. MA contributed to the discussion. CS reviewed the literature and references. All authors read and approved the final manuscript.
